# Efficacy of Topical Phenytoin in the Management of Diabetic Foot Ulcers: A Systematic Review

**DOI:** 10.7759/cureus.87517

**Published:** 2025-07-08

**Authors:** Charles P Barry, Mohamed Shefan S Hameed, Abilash Sathyanarayanan

**Affiliations:** 1 Endocrinology and Diabetes, Nottingham University Hospitals NHS Trust, Nottingham, GBR; 2 Internal Medicine, Nottingham University Hospitals NHS Trust, Nottingham, GBR

**Keywords:** diabetic foot ulcer (dfu), diabetic foot ulcer management, foot ulcer, phenytoin (pht), topical phenytoin, ulcer healing

## Abstract

Phenytoin has been used as an anti-seizure medication since 1939. There has been interest in its healing properties, with many randomized controlled trials (RCTs) investigating and recommending its use in diabetic foot ulcer (DFU) disease. Despite this, the International Working Group on the Diabetic Foot (IWGDF) does not support its use. The objective of this systematic review was to assess the use of phenytoin in the treatment of DFU disease compared to a control. Systematic searches of Medline, Embase, Google Scholar, the Cochrane Library, and ClinicalTrials.gov were conducted on January 5, 2024, using the keywords "phenytoin", "diabetes", "ulcer", and "RCT". Methodological quality was assessed using the Risk of Bias 2 (RoB 2) tool. A total of 23 studies were included in the systematic review, of which 20 recommended the use of phenytoin. However, the quality of the studies was poor. Of the included studies, 17 failed to implement blinding, 10 did not describe the randomization process, and only one completed a sample size calculation. There was a wide variation in both the study duration and the outcomes chosen to report the change in ulcer size. For this reason, a meta-analysis could not be performed. The current evidence does not support the use of phenytoin in DFU disease; further studies with larger patient numbers and reduced risk of bias are needed.

## Introduction and background

Diabetic foot ulcer (DFU) disease is one of the most common complications of diabetes, with estimates suggesting up to one-third develop a foot ulcer during their lifetime [1]. DFUs are the most common cause of lower limb amputation, a significant cause of morbidity and mortality for those with diabetes [2]. The yearly cost in England is estimated to be between £837 and 962 million; in the US, this estimate reaches $9-13 billion [3-4]. Worldwide, the prevalence of diabetes is increasing; this rise is estimated to be greater in low- and middle-income countries (LMIC) where resources and specialized services are more constrained [5]. The management of DFUs requires a multidisciplinary approach involving assessment at specialist foot care services. If severe, systemic antibiotics and imaging may be required. Surgical intervention is considered if signs of ischemia or necrosis are present [6]. Amongst topical therapies, except for sucrose-octasulfate dressings, the evidence for other compounds is poor. This is the case for antimicrobials, antiseptics, and phenytoin.

Phenytoin has been used as an anti-epileptic medication since 1939 [7]. Gingival hypertrophy was a recognized side effect, which generated the hypothesis that it could promote healing throughout the body [8,9]. The mechanism is not yet known, but studies suggest that it may promote collagen deposition, fibroblast migration, and angiogenesis [10,11]. Importantly, systemic absorption of phenytoin is not significant with topical use [12]. For these reasons, as well as its simple application and low cost, phenytoin has long been proposed as an agent that could promote healing in DFU disease. Many randomized controlled trials (RCTs) have investigated this with positive results. However, poor study design and high risk of bias have meant there is insufficient evidence for the International Working Group on the Diabetic Foot (IWGDF) to recommend topical phenytoin [13]. Prior systematic reviews have formed similar conclusions [13-15]. However, this area continues to attract interest, with at least one RCT being released every year for the last 10 years. This systematic review is the largest and most up-to-date evaluation of phenytoin use in DFU disease compared to a control.

## Review

This systematic review is reported in accordance with the recommendations of the Preferred Reporting Items for Systematic Reviews and Meta-Analyses (PRISMA) guidelines [16]. The predefined protocol was registered with the International Platform of Registered Systematic Review and Meta-analysis Protocols (INPLASY) with the registration number INPLASY202410023.

Eligibility criteria

The Patient, Intervention, Control, Outcome, and Study type (PICOS) tool was used to create the inclusion criteria: patients were those with diabetes-related foot ulcers, the intervention was phenytoin, the control included standard care or placebo, outcomes were identified as all healing-related outcomes, and the study type was RCTs.

Exclusion criteria included the following: (1) ulcers of other aetiologies, such as pressure ulcers or leprosy ulcers, and (2) comparative studies, systematic reviews, editorial letters, or case reports.

Selection of studies

The search was conducted independently by two authors using the following databases: Medline, Embase, Cochrane Library, ClinicalTrials.gov, and Google Scholar. The search strategies are outlined in the appendix. Databases were searched on January 5, 2024. Duplicates were removed. Two authors independently screened the studies against the eligibility criteria, first by the title and then by the abstract. Full texts were then retrieved. Any discrepancies were resolved by a third author.

Data extraction

Data extraction was carried out independently by two authors. This included the following variables: all healing-related outcomes and whether these were statistically significant, the number of patients, treatment arm details, ulcer duration at randomization, inclusion of vascular disease, blinding and allocation concealment, and study duration. Data were filled directly into two separate tables, which were then collated into one.

Data analysis

Data were analyzed descriptively due to their heterogeneity. Outcomes were grouped into similar categories for descriptive analysis. A meta-analysis was not completed (reasons explained below).

Quality assessment

The quality of each paper was independently assessed by two authors using the Risk of Bias 2 (RoB 2) tool [17]. Any discrepancies were resolved by the third author. The results were collated, and then the Robvis visualization tool was used to create the summary diagram [18].

Results

**Figure 1:**
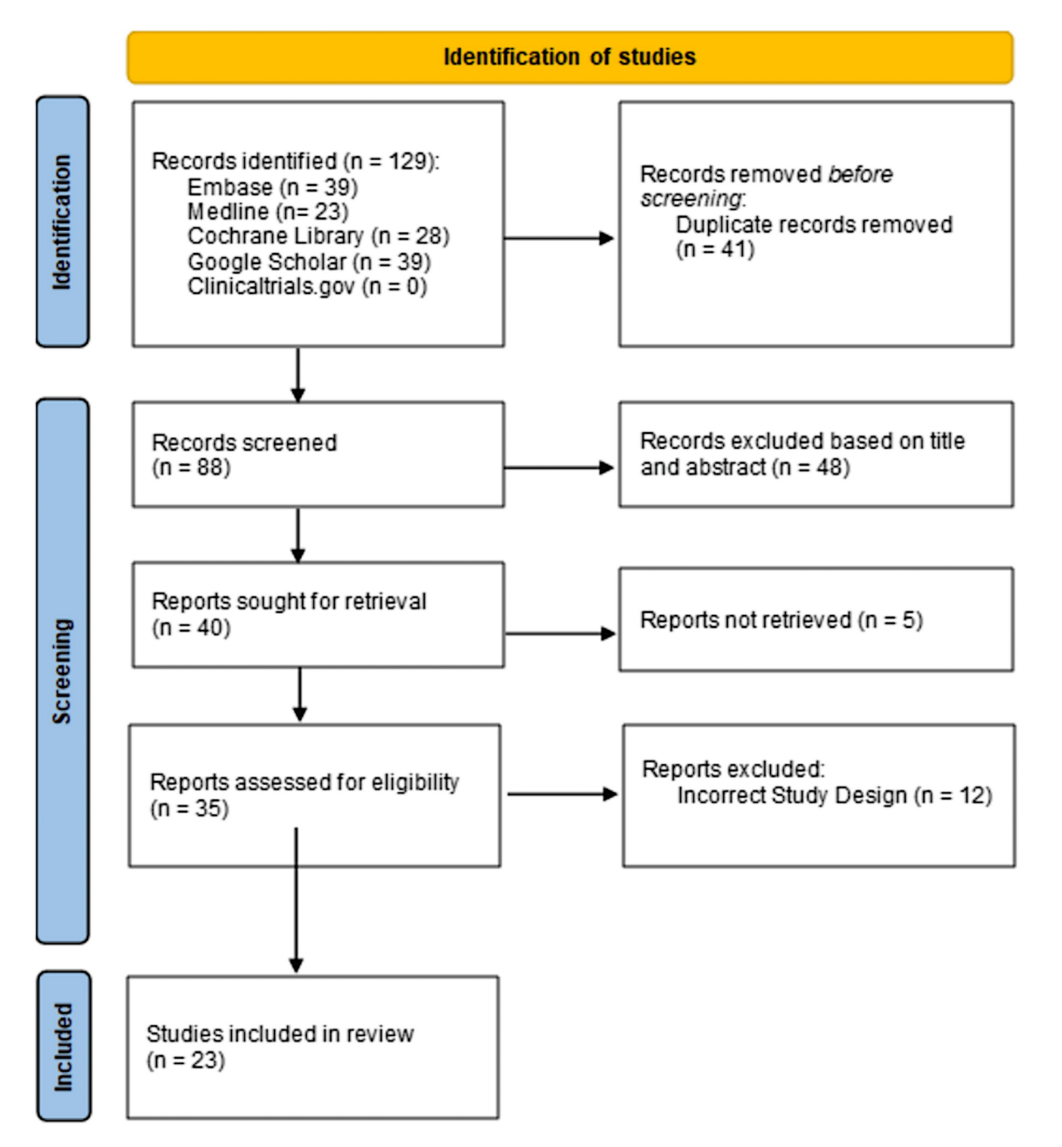
PRISMA flowchart for study identification PRISMA: Preferred Reporting Items for Systematic Reviews and Meta-Analyses

A total of 129 records were obtained from the systematic search. Studies were organized according to the inclusion and exclusion criteria discussed above. Following the removal of 41 duplicates and the screening of titles and abstracts, 48 additional studies were removed. This left 40 studies for full-text evaluation; five studies were not retrieved, and 12 were excluded due to incorrect study design. This left 23 studies for inclusion in the systematic review [19-41], which is depicted in Figure 1. Table 1 provides the characteristics of the included studies.

**Table 1:** Characteristics of the included studies *: Indicates a finding (to support the beneficial effect of phenytoin) that was statistically significant (p < 0.05) as reported. It does NOT infer a methodologically reliable or clinically relevant effect. §: No information reported. †: If no explicit mention of excluding patients with vascular disease, it was assumed they were included. ‡: Primary outcome in BOLD (majority of studies did not identify a primary outcome). ^: Sample size was calculated.

First Author (Year, Country)	Total Participants (Phenytoin, Control)	Phenytoin Arm	Control Arm	Ulcer Duration at Randomization (Weeks)	Vascular Disease^†^	Blinding/Allocation Concealment	Study Duration (Weeks)	Outcome Measures/Results
Ahmed [19] (2014, Pakistan)	60 (30, 30)	Phenytoin powder	Normal saline, povidone iodine, Vaseline	4	Excluded	§/lottery method	8	(1) Mean percentage reduction in ulcer area*, (2) Efficacy (>50% reduction in area) in N*, (3) Ulcer area baseline & study end
Azeez [20] (2017, India)	90 (45, 45)	Phenytoin powder	Povidone iodine	0	Excluded	§	2	(1) Mean rate of granulation tissue formation*, (2) Mean duration of hospital stay*, (3) Number of days required for healing, (4) Bacterial culture (positive or negative), (5) Side effects of topical phenytoin, (6) Skin graft uptake*
Bharadva [21] (2017, India)	56 (28, 28)	Phenytoin suspension	Povidone iodine	0	Included	§	2	(1) Wound discharge reduction*, (2) Slough reduction*, (3) Mean granulation tissue (pale/normal/absent)*, (4) Mean duration of hospital stay*, (5) Organisms isolated from the wound (6) Negative culture sensitivity
Gunasekaran [22] (2017, India)	60 (30, 30)	Phenytoin suspension	Normal saline	0	Excluded	§/computer randomized	3	(1) Negative culture percentage*, (2) Rate of granulation tissue formation*, (3) Ulcer surface area reduction, (4) Mean duration of hospital stay
Hajong [23] (2016, India)	100 (50, 50)	Phenytoin suspension	Normal saline	0	Included	§/computer randomized	2	(1) Time to 50% wound contracture area*, (2) Epithelialization time*, (3) Epithelialization time by grade of ulcer
Jayalal [24] (2015, India)	60 (30, 30)	Phenytoin suspension	Povidone iodine, saline wash, magnesium sulfate	4	Excluded	Observer-blind/lottery method	1–3	(1) Mean percentage surface area reduction*, (2) Number with >50% reduction in surface area*, (3) Number with granulation tissue present*, (4) Slough reduction*, (5) Mean duration of hospital stay*, (6) Pain (visual analogue scale)*
Kalyani [25] (2022, India)	30 (10, 10, 10)	Phenytoin	(1) Normal wound dressing, (2) topical insulin	0	Included	§	4	(1) Mean percentage reduction of wound area, (2) Mean percentage reduction of wound depth*
Motawea [26] (2019, Egypt)	27 (9, 9, 9)	(1) Phenytoin nanostructured lipid carrier hydrogel, (2) Phenytoin hydrogel	Blank hydrogel	0	Excluded	Double-blind/block design	8	(1) Mean percentage reduction in wound area*, (2) Mean wound depth*, (3) Percentage wound healing*
Muthukumarasamy [27](1991, India)	100 (50, 50)	Phenytoin powder	Occlusive dressing	0	Excluded	§	5	(1) Mean percentage reduction in ulcer area*, (2) Sough reduction*, (3) Negative wound cultures*, (4) Time to healthy granulation tissue*, (5) Time to complete healing*, (6) Clinical improvement*, (7) Wound discharge reduction
Nagaraj [28] (2022, India)	60 (20, 20, 20)	Phenytoin	(1) Insulin injected into wound, (2) normal saline dressing	0	Included	§/number lot approach	4	(1) Mean number of days for healing, (2) Mean difference in wound area before and after, (3) Mean difference in wound depth before and after, (4) Number of patients with granulation tissue (poor/satisfactory/healthy)
Pai [29] (2001, India)	70 (36, 34)	Phenytoin powder	Talcum powder and colloidal silicon dioxide	0	Included	Double-blind/§	6	(1) Mean percentage reduction in ulcer area, (2) Time to disappearance of wound discharge, (3) Number of ulcers with negative wound swabs, (4) Quality of granulation tissue
Patil [30] (2013, India)	100 (50, 50)	Phenytoin suspension	Normal saline	0	Excluded	Observer-blind/lottery method	6	(1) Wound discharge reduction*, (2) Slough reduction*, (3) Granulation tissue quality (absent/pale/good/absent/pale/good)*, (4) Mean duration of hospital stay*
Prabhu [31] (2017, India)	97 (49, 48)	Phenytoin & metronidazole powder	Metronidazole powder	4	Included	Double-blind/block randomization	2	(1) Mean percentage reduction in ulcer area*, (2) Percentage increase in granulation tissue*, (3) Number of negative cultures, (4) Percentage of graft uptake*
Prasad [32] (2017, India)	50 (25, 25)	Phenytoin suspension	Povidone iodine	0	Included	§/lottery method	2	(1) Mean ulcer area*, (2) Rate of granulation tissue formation (as a percentage of ulcer area)*, (3) Negative wound culture*, (4) Mean duration of hospital stay, (5) Graft uptake*
Reddy [33] (2021, India)	60 (30, 30)	Phenytoin suspension	Normal saline	0	Excluded	§/computer randomized	4	(1) Mean wound area reduction* and (2) culture growth between day 0 & day 10*
Sandhu [34] (2017, India)	40 (20, 20)	Phenytoin suspension	Povidone iodine	0	Included	§	2	(1) Negative pus culture*, (2) Mean percentage of ulcer area covered by granulation tissue, (3) Mean duration of hospital stay, (4) Graft uptake*
Selvaraj [35] (2016, India)	100 (50, 50)	Phenytoin suspension	Povidone iodine	0	Excluded	§	2	(1) Mean percentage of ulcer area with granulation, (2) Mean ulcer surface area, (3) Mean duration of hospital stay, (4) Percentage negative culture, (5) Graft uptake
Shaw [36] (2011, UK)	65 (29, 27)^^^	Phenytoin-containing alginate dressing	Alginate dressing	4	Excluded	Double-blind/independently randomized	16	(1) Time to healing (survival analysis), (2) Percentage change in wound area, (3) Ulcers with positive wound swabs, (4) Serum phenytoin levels (<2.2mg/L), (5) Pain (McGill Pain Questionnaire & VAS)
Silodia [37] (2018, India)	50 (25, 25)	Phenytoin suspension	Povidone iodine	0	Excluded	§	2	(1) Number of ulcers with healthy granulation*, (2) Mean reduction in ulcer area*, (3) Number with >50% reduction in ulcer area*
Soundarapandiyan [38](2017, India)	100 (50, 50)	Phenytoin suspension	Povidone iodine, magnesium sulfate dressing	0	Included	§/lottery method	2	(1) Percentage of ulcers with healthy granulation*, (2) Mean percentage reduction in ulcer area*, (3) Mean duration of hospital stay
Sreehita [39] (2023, India)	75 (25, 25, 25)	Phenytoin-injected dressing	(1) Sucralfate syrup dressing, (2) hydrogel cream dressing	0	Excluded	§	10	(1) Mean reduction in ulcer area week 4 & week 10, (2) Mean duration of hospital stay, (3) Abstinence from work, (4) Complete/Partial Healing
Sudhir [40] (2020, India)	70 (35, 35)	Phenytoin suspension	Povidone iodine	2	Excluded	§	2	(1) Number of ulcers with wound discharge*, (2) Number of ulcers with slough*, (3) Number of ulcers with good granulation*, (4) Mean percentage reduction in ulcer area day 7 & 14, (5) Mean duration of hospital stay*, (6) Mean number of dressings*
Vardhan [41] (2016, India)	70 (35, 35)	Phenytoin suspension	Povidone iodine	0	Included	§	2	(1) Mean duration of hospital stay*, (2) Patients with healthy granulation tissue*

Quality Assessment

**Figure 2:**
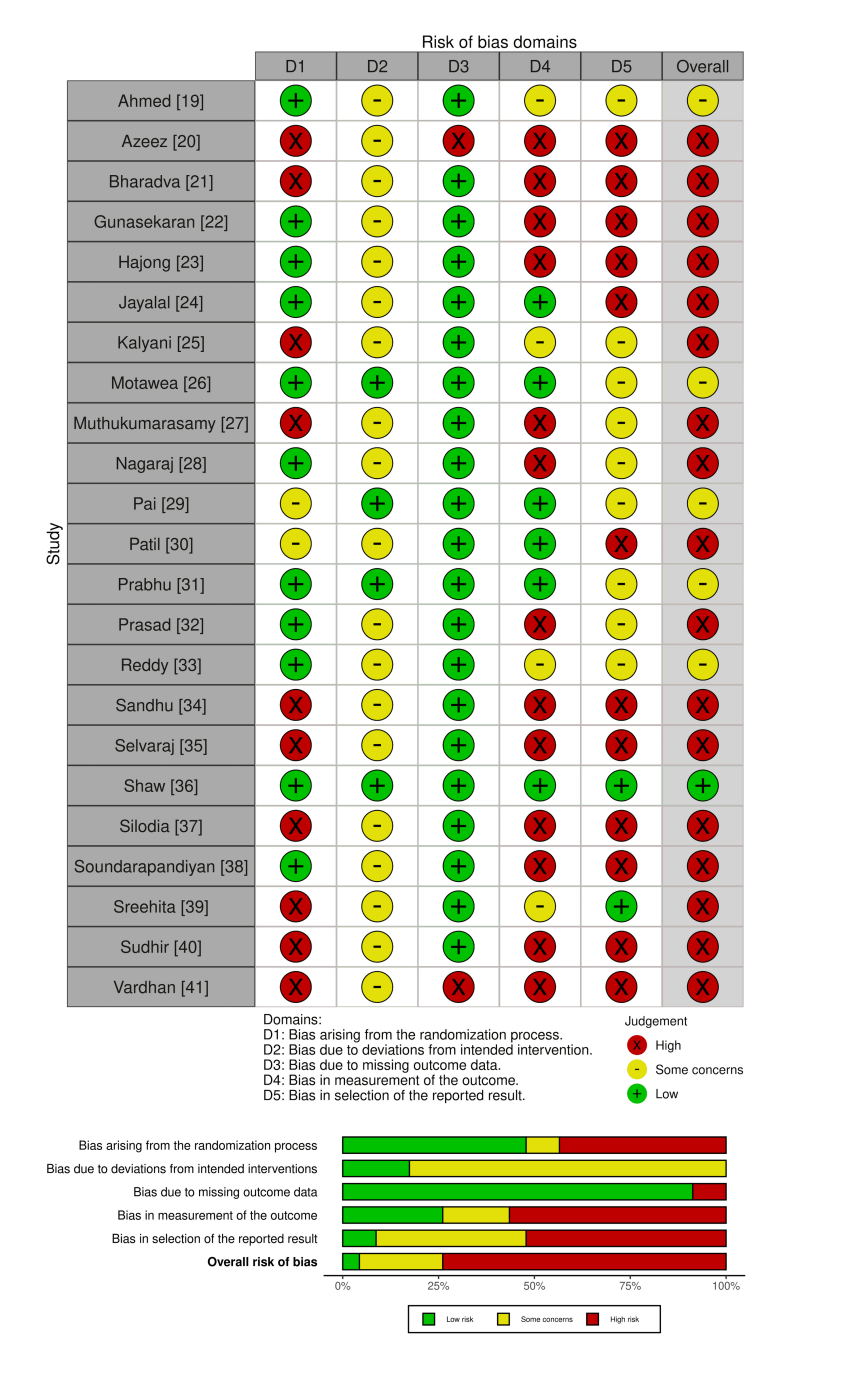
Traffic light and summary plot summarizing the quality assessment of the included studies

Overall, the quality of the studies was low, with 17 studies rated as having a high risk of bias and only one achieving a low risk. Ten studies did not adequately describe their randomization process. Only six studies had blinded assessors, and only four of those had blinded participants. Outcomes chosen for assessing healing varied significantly between the studies. Two studies lacked explanations for the missing outcome data, as summarized in Figure 2.

Interventions

Phenytoin was either delivered as a powder applied directly to the ulcer or as a suspension (dissolved in normal saline) that the dressings were soaked in. The dose of phenytoin used was based on the ulcer surface area for all studies.

Regarding control interventions, 14 studies used povidone iodine or normal saline [19-23,28,30,32-35,37,40,41]. Prabhu et al. used metronidazole powder, administered with phenytoin for the active intervention [31]. As both are powders, this ensured blinding was maintained when both the control and active intervention were delivered. Other control interventions included magnesium sulfate, hydrogel, occlusive dressings, talcum powder/colloidal silicon dioxide, and alginate dressing. Four studies included other treatment arms investigating injected insulin, sucralfate syrup dressing, and phenytoin nanostructured lipid carrier hydrogel; these outcomes have not been investigated as they are beyond the scope of this systematic review.

Outcomes

Quantification of the ulcer healing was assessed in three ways: ulcer area change, ulcer depth change, or time taken for ulcer closure. A total of 17 papers reported area change before and after, and 10 reported a significant difference between phenytoin and control. Wound depth change was reported by three papers, where two studies reported a significant difference. The time to complete healing was reported by three papers, with one finding a significant difference.

Hallmarks of healing were also used to assess the role of phenytoin in the healing process. These hallmarks include the quantification of granulation tissue, the absence of slough, the absence of wound discharge, a negative wound culture, and graft uptake. A total of 17 papers reported a change in granulation tissue, but the methods of measuring this varied, including the rate of formation, number with healthy or good granulation, time to healthy granulation, or percentage of the wound covered by granulation (of which 13 papers reported a significant positive effect in these outcomes). Five papers reported the absence of slough; all five were significant. Five papers reported a change in wound discharge; three were significant. A total of 11 papers reported a difference in wound swab culture results between phenytoin and the control; five of these differences were significant. Graft uptake was reported by five papers, and four papers reported improved graft uptake with phenytoin.

Other outcomes unrelated to healing included assessments of pain scores and the duration of hospital stay or time away from work. Two papers reported pain scores as an outcome; one reported a significant difference in pain scores between the phenytoin and control groups. The duration of hospital stay was reported by 12 papers; six papers reported reduced hospital stay with phenytoin compared to controls.

Discussion

The aim of this systematic review was to investigate the effect of phenytoin on DFU disease by examining RCTs comparing phenytoin to standard care/placebo. A total of 23 RCTs were identified, with 20 studies conducted in India and one each in Egypt, Pakistan, and the United Kingdom. Overall, 20 of the 23 studies reported results that recommended the use of phenytoin based on various outcomes, including an improved healing area, increased presence of granulation tissue, and absence of slough. However, these results need to be interpreted with caution.

Firstly, the quality of the studies was very poor. For example, 10 studies did not describe the randomization process, and 17 failed to implement blinding, including a lack of blinding in outcome assessment. Fundamental lapses in the randomization and blinding process mean that the risk of systematic error is high, leading to significant biases in these conclusions.

Secondly, there is a high risk of random error in these conclusions. Only one study described an appropriate a priori sample size calculation, and only four identified a primary endpoint for analysis. This introduces a high risk of bias due to random error and a potential for false positives resulting from multiple testing.

Thirdly, any attempt to minimize the above by performing a meta-analysis of the studies was not possible due to marked heterogeneity between the studies and non-compliance with current reporting standards. The main sources of heterogeneity were the time of randomization (ranging from ulcers at first presentation to 16 weeks), differences in the total duration of the active study period, the inclusion of ulcers with underlying vascular disease, and marked variation in the outcome measure. This was also expressed by Shaw et al. in their 2008 investigation of phenytoin use [14].

Finally, ulcer size data could theoretically be used to perform a meta-analysis to obtain an overall size effect by grouping studies with similar characteristics. However, the papers report ulcer sizes in various metrics (e.g., mean reduction in size, absolute sizes at the end of the study, and ulcer measurements at different time points), which limit the statistical feasibility of undertaking such a calculation. Furthermore, despite contacting various authors for raw data on ulcer sizes, we were unable to obtain the required information to conduct a reliable meta-analysis.

The results of this systematic review reaffirm the conclusions from the IWGDF guidelines, which do not recommend the use of phenytoin due to the low certainty of the evidence. Both Shaw et al. and Vas et al. also highlighted the varying methodological quality of the studies [14,15].

The strength of this study is that it is the most up-to-date systematic review of trials investigating phenytoin in DFU disease. The inclusion of multiple new studies during our review indicates a continued interest in this area. This systematic review also summarizes various outcomes that may serve as surrogate endpoints in future clinical trials, especially when designing multi-stage or multi-arm clinical trials. This will guide future researchers in planning their trials more efficiently.

The main limitation of our review is that, given the poor reporting in these studies and multiple positive results, there is likely to be significant publication bias despite a broad inclusion criterion for the review. In addition, there is a high regional skew in the origin of these studies (India is the main contributor); this may reflect a local interest in the intervention based on clinical context; however, the reason for this needs to be explored by funding organizations in the future.

## Conclusions

In conclusion, many of the studies have recommended the use of phenytoin, with results suggesting that it has a beneficial effect on wound healing. However, the absence of blinding, insufficient randomization, and lack of sample size calculation indicate a high risk of bias. There was also marked heterogeneity between the studies, with varying durations, reported size metrics, and times of randomization. At present, the available evidence does not support the use of phenytoin. The results should be treated as hypothesis-generating conclusions. Further studies are required, with larger patient numbers and minimized risk of both systematic and random errors.

## Appendices

Search strategies

Search strategies for Medline, Embase, and Cochrane Library (CENTRAL) are listed below. The Medline search strategy is demonstrated in Table 2, the Embase search strategy is demonstrated in Table 3, and the Cochrane Library (CENTRAL) search strategy is demonstrated in Table 4. The search strategy for ClinicalTrials.gov involved using the term "Diabetes" for the "Condition/Disease" category, "Ulcer" for the "Other Terms" category, and "Phenytoin" for the "Intervention/Treatment" category. The Google Scholar search strategy involved completing a search using the three search terms "Diabetes, Phenytoin, Ulcer". The titles of the first 100 pages were assessed for eligibility (1000 publications).

**Table 2:** Medline search strategy

Line Number	Term
1	randomized controlled trial.pt.
2	controlled clinical trial.pt.
3	randomized.ab.
4	placebo.ab.
5	drug therapy.fs.
6	randomly.ab.
7	trial.ab.
8	groups.ab.
9	1 or 2 or 3 or 4 or 5 or 6 or 7 or 8
10	exp animals/not humans.sh.
11	9 not 10
12	phenytoin.mp. or Phenytoin/
13	Ulcer/or ulcer.mp.
14	Diabetes Mellitus/or diabetes.mp.
15	Diabetic Retinopathy/or Diabetic Ketoacidosis/or diabetic.mp. or Diabetic Foot/or Diabetic Nephropathies/or Diabetic Cardiomyopathies/or Diabetic Angiopathies/or Diabetic Neuropathies/
16	14 or 15
17	wound.mp. or ‘Wounds and Injuries’/
18	13 or 17
19	11 and 12 and 16 and 18

**Table 3:** Embase search strategy

Line Number	Term
1	exp randomized controlled trial/
2	Controlled clinical trial/
3	random$.ti,ab.
4	randomization/
5	intermethod comparison/
6	placebo.ti,ab.
7	(compare or compared or comparison).ti.
8	((evaluated or evaluate or evaluating or assessed or assess) and (compare or compared or comparing or comparison)).ab.
9	(open adj label).ti,ab.
10	((double or single or doubly or singly) adj (blind or blinded or blindly)).ti,ab.
11	double blind procedure/
12	parallel group$1.ti,ab.
13	(crossover or cross over).ti,ab.
14	'((assign$ or match or matched or allocation) adj5 (alternate or group$1 or intervention$1 or patient$1 or subject$1 or participant$1)).ti,ab.'
15	(assigned or allocated).ti,ab.
16	(controlled adj7 (study or design or trial)).ti,ab.
17	(volunteer or volunteers).ti,ab
18	human experiment/
19	trial.ti.
20	or/1-19
21	'(random$ adj sampl$ adj7 (“cross section$” or questionnaire$1 or survey$ or database$1)).ti,ab. not (comparative study/or controlled study/or randomi?ed controlled.ti,ab. or randomly assigned.ti,ab.)'
22	Cross-sectional study/not (exp randomized controlled trial/or controlled clinical study/or controlled study/or randomi?ed controlled.ti,ab. or control group$1.ti,ab.)
23	(((case adj control$) and random$) not randomi?ed controlled).ti,ab.
24	Systematic review.ti,ab. not (trial or study).ti.
25	(nonrandomi$ not random$).ti,ab.
26	“random field$”.ti,ab.
27	(random cluster adj3 sampl$).ti,ab.
28	(review.ab. and review.pt.) not trial.ti.
29	“we searched”.ab. and (review.ti. or review.pt.)
30	“update review”.ab.
31	(database adj4 searched).ab.
32	(rat or rats or mouse or mice or swine or porcine or murine or sheep or lambs or pigs or piglets or rabbit or rabbits or cat or cats or dog or dogs or cattle or bovine or monkey or monkeys or trout or marmoset$1).ti. and animal experiment/
33	Animal experiment/not (human experiment/or human/)
34	or/21-33
35	20 not 34
36	diabetes.mp. or diabetes mellitus/
37	diabetic.mp. or diabetes mellitus/
38	phenytoin.mp. or phenytoin/
39	ulcer.mp. or ulcer/
40	wound.mp. or wound/
41	36 or 37
42	39 or 40
43	35 and 38 and 41 and 42

**Table 4:** Cochrane Library (CENTRAL) search strategy

Line Number	Term
1	diabetes
2	diabetic
3	phenytoin
4	ulcer
5	wound
6	1 or 2
7	4 or 5
8	6 and 7 and 3
